# Influence of spin finish on degradation, functionalization and long-term storage of polyethylene terephthalate fabrics dedicated to ligament prostheses

**DOI:** 10.1038/s41598-021-83572-8

**Published:** 2021-02-19

**Authors:** Tuan Ngoc Nguyen, Andre Rangel, David W. Grainger, Véronique Migonney

**Affiliations:** 1 Chemistry, Structures and Properties of Biomaterials and Therapeutic Agents Laboratory, LBPS-CSPBAT, UMR CNRS 7244, Université Sorbonne Paris Nord, Villetaneuse, France; 2 Department of Biomedical Engineering, and Department of Pharmaceutics and Pharmaceutical Chemistry, University of Utah, Salt Lake City, UT USA

**Keywords:** Biomaterials, Materials for devices, Surface chemistry

## Abstract

Polyethylene terephthalate (PET) fibers and fabrics are widely used for medical device applications such as vascular and anterior cruciate ligament prostheses. Several years ago, we began functionalizing PET fabrics using anionic polymers to enhance their biocompatibility, cell adhesion, proliferation and functional performance as PET ligament prostheses. Polymer functionalization followed a grafting-from process from virgin PET surfaces subject to spin-finish oil additive removal under Soxhlet extraction to remove residual fiber manufacturing oil. Nevertheless, with increasing time from manufacture, PET fabrics stored without a spin finish removal step exhibited degradation of spin finish oil, leading to (1) incomplete surface cleaning, and (2) PET surface degradation. Moreover, oxidizing agents present in the residual degraded oil prevented reliable functionalization of the prosthesis fibers in these PET fabrics. This study compares effects of PET fabric/spin finish oil storage on PET fabric anionic polymer functionalization across two PET fabric ligament storage groups: (1) 2- and 10- year old ligaments, and (2) 26-year old ligaments. Strong interactions between degraded spin finish oil and PET fiber surfaces after long storage times were demonstrated via extraction yield; oil chemistry changed assessed by spectral analysis. Polymer grafting/functionalization efficiency on stored PET fabrics was correlated using atomic force microscopy, including fiber surface roughness and relationships between grafting degree and surface Young’s modulus. New PET fabric Young’s modulus significantly decreased by anionic polymer functionalization (to 96%, grafting degree 1.6 µmol/g) and to reduced modulus and efficiency (29%) for 10 years storage fabric (grafting degree ~ 1 µmol/g). As fiber spin finish is mandatory in biomedically applicable fiber fabrication, assessing effects of spin finish oil on commercial polymer fabrics after longer storage under various conditions (UV light, temperature) is necessary to understand possible impacts on fiber degradation and surface functionalization.

## Introduction

Medical grade polyethylene terephthalate (polyester, PET) yarns and fibers used for diverse medical applications, including vascular and ligament prosthetic grafts, are distinct in several aspects from PET used for other non-medical textile applications. Notably, PET strength and fiber structures are deliberately altered for implantable medical applications to produce high strength, high modulus and low elongation materials for device uses. Polymer technology, textile and spinning techniques, drawing methods and processing machinery have been critical to enabling new PET fiber and fabric processing that yield fibers with these requisite properties^1–3^.

Given current broad use of PET fibers and fabrics for medical devices applications^4^, the PET ligament prosthesis holds a unique place. Despite clinical prominence of PET woven vascular prostheses for many years, recognized as the most reliable solution for replacing diseased vessels^5^, PET ligament prostheses are more recently introduced and clinically important^6^. Ligament tears are increasingly common injuries in pivot sports, requiring stabilization of the knee by surgical ligament reconstruction to prevent osteoarthritis. Basically, these prostheses facilitate tissue re-connection of the torn injured ligament component, involving reconstruction of new ligament through host cellular re-attachment and healing^7,8^. This requires extended rehabilitation time. Nonetheless, textile ligament prostheses use remains limited due to poor control of in vivo degradation risk factors of implanted prostheses and performance that is sub-standard versus clinical standards of care. Improved ligament prostheses designs are sought.

After disastrous clinical results for the first generation of artificial ligaments, largely based on PET textile biomaterials^6^, second- and third- generation PET ligament prostheses have yielded improvements in both materials and implant designs. Second-generation PET implants, developed by LARS laboratories (France), focused on a biomaterial prosthesis structure mimicking that of the natural ligament^9^. Third-generation designs exploited further surface functionalizing of the PET ligament prosthesis with grafted bioactive polymers to improve biocompatibility and tissue “bio-integration”, i.e. increased host cell adhesion, proliferation and signaling to stabilize implant integration^10,11^. Migonney et al. showed that poly(sodium 4-styrene sulfonate) (PNaSS) grafting onto PET surfaces improves both adhesion and functions of fibroblast cells that constitute important ligament and tendon endogenous cells^10–14^.

PET ligament prostheses comprise complex knitted and woven fabrics made from commercial PET fibers previously coated with an added manufacturing oil by design^7^. The process called “spin-finish” is performed using FDA-approved^15^ synthetic or natural oil for biomedical applications^16^. Indeed, spin finish oil is coated to protect fibers, reduce fiber and fabric adhesion and stickiness, improve handling and assembly, and provide optimal properties that enable extrusion of precision calibrated PET fiber dimensions. It is well-recognized that fiber spin finish oil allows PET fabrics to be easily woven, handled and safely stored^16,17^.

Spin finish treatments of fibers dedicated to medical devices use oils such as soybean oil containing fatty acids ester and epoxide groups for antioxidant properties^18–22^. However, depending on storage conditions (i.e. short or long term, ambient or controlled atmosphere, and strictly controlled or uncontrolled temperature), these spin finishing (or “sizing”) oils either remain inert or degrade, generating oxidized species and radicals that can affect polymer fiber integrity and impede their further functionalization and properties. Prior to surface functionalization of polymers fibers or fabrics, a surface pre-treatment called spin finish removal (SFR) is usually performed using Soxhlet solvent extraction to remove the protective spin finish oil^23^. Reliable SFR is required for PNaSS “grafting from” reactions on clean PET fiber surfaces through radical grafting polymerization processes^10–14^.

Rahman and East reported spin finish effects on the hydrolysis of medical-grade PET fibers used to produce antithrombotic fabric vascular grafts^23^. They demonstrated that spin-finish oil reduced PET degradation by ambient oxidation and hydrolysis. However, no reports describe any effect of finish oil and storage on SFR pre-treatment of PET for surface functionalization.

This study was designed to determine the influence of spin-finish treatment and long-term storage on PET fabric degradation and functionalization required for production of PET ligament prostheses. SFR on 2-, 10-, and 26-year old PET ligament fibers from storage was first achieved using diethyl ether (DE) as solvent for oil extraction in a Soxhlet system. Further experiments compared the PET fiber extraction efficiencies of other solvents (DE vs. n-hexane, and tetrahydrofuran, THF) with spin finish oil and resulting PET fiber alterations. After these assays, PET fabric functionalization was assessed following PNaSS radical “grafting from” polymer coating techniques in order to evaluate the efficiency and effects of the SFR process on polymer surface grafting. Results demonstrate that spin finish oil instability over time and extended PET fiber storage conditions alters SFR, surface functionalization and PET fiber/fabric degradation under long-term storage. A mechanism is proposed that correlates the significant and deleterious interactions between spin finish oil and PET fabric interfaces during storage, degrading PET surfaces and reducing PNaSS GR after SFR and grafting functionalization processing. These results are important to understand and control storage and shelf-life conditions affecting critical properties for PET fiber-based medical implants.

## Materials

Polyethylene terephthalate (PET) fabrics comprising knitted and woven fibers of 25 µm diameter were kindly provided by LARS (Paris, France) and stored at 20 °C under ambient atmosphere for different times: 26 years (PET_1993_), 10 years (PET_2009_), and 2 years (PET_2018_). Diethyl ether (DE) (Fisher), n-hexane (Fisher), and tetrahydrofuran (THF, Fisher) were used as extraction solvents. PET film (as reference for FTIR and DSC controls, semi-crystalline PET film, ES301450, 0.25 mm thickness, no batch or lot or production date evident) was purchased from Goodfellow (Paris, France).

### Fabric preparation

PET fabrics were cut into small pieces (3 × 3 cm, 35–40 mg). These samples were (1) DE washed by Soxhlet extraction to achieve the SFR process, (2) quickly dried by DE evaporation, (3) rinsed in ethanol, and (4) dried under vacuum at room temperature for 1 h before use. The PET fabric after extensive SFR (“virgin PET fabric”) was also used as reference sample for comparative analysis.

### Spin finish oil preparation

Two spin finish oils were investigated in this study: (1) spin-finish epoxidized soybean oil (SFSB-oil, Sigma Aldrich, virgin oil comparator, CAS no. 8013-07-8) (Fig. A.1, supplementary information) and (2) spin finish oil extracted from stored commercial PET fabrics (PET_1993_, PET_2009_ and PET_2018_) by Soxhlet extraction processing (SF-Ext).

SFSB-oil has been used for spin finish treatment of Lars commercial PET_2009 and 2018_ fibers. In contrast, the spin finish oil used for PET_1993_ was not identified and is no longer used.

### Purification of sodium (4-styrene sulfonate) (NaSS)

Monomer NaSS was purified by a re-crystallization process^11,24^ as follows: 90 g of NaSS was dissolved in a mixture of ethanol and distilled water (9:1, v/v) and heated at 70 °C for 10 h. The solution was then filtered and left to re-crystallize at 4 °C for 24 h. Re-crystallized NaSS monomer was collected and dried under vacuum at 30 °C for 6 h. The product was kept at 4 °C protected from light.

## Methods

### Spin finish removal (SFR) process

Soxhlet extraction was performed at each respective solvent boiling point (n-hexane, THF or DE) to remove the spin finish oil layer from PET fabrics for 6 h, using 60 mL solvent per 0.1 g fabric samples. Solvent recovery was around 60–75% v/v. The spin finish extraction yield (*E*) was calculated as follows:

$$ E = \frac{{m_{0} - m_{e} }}{{m_{0} }} \times 100 $$

where m_0_ is the initial mass of the PET/oil sample; m_e_ is the mass of the PET sample after solvent extraction in the spin finish removal process.

### Collection and degradation analysis of spin finish oils

#### Collecting extracted spin finish oil from fibers/fabrics

Extracted DE solutions of SF oil were (1) collected by Soxhlet extractions of the different PET fabrics (PET_1993,_ PET_2009_ and PET_2018_), (2) evaporated using a rotary evaporator (LabTech EV311H) for 40 min, 40 rpm water bath at 30 °C, (3) dried under vacuum and weighed until constant mass weight of the resulting oil extract residue.

#### Spin finish oil degradation analysis

Radical and/or oxidative chemistry propagation to and cross-linking and artificially degraded by (1) UV radiation (UV 6.C, 6 W, 254 nm wavelength, sample distance 140 mm from the lamp, irradiation 1.75 mW/cm^2^) or (2) heating at 100 °C for 10 days, both in air (ambient atmosphere). After UV or thermal degradation, the resulting SFSB oil samples were analyzed using FTIR spectroscopy and their solubility in diethyl ether (DE) was assessed using visual observation of liquid phase separation (Fig. A.2, supplementary information).

### Poly (4-styrene sulfonate) grafting on PET after ozonation

Poly(4-styrene sulfonate) (PNaSS) grafting of PET fabrics consists of a two-step process^11,24–27^: (1) fiber ozone oxidation (ozonation) and (2) monomer radical-initiated thermal grafting (polymerization/grafting from). This process was performed on each solvent-extracted PET fabric sample (3 cm × 3 cm).

1. *Ozonation* three PET fabric samples were placed in a glass reactor in which ozone gas (generator BMT 802 N, 0.6 bar, 0.6 L/min) was left bubbling in water for 10 min at 30°C^11,24^. The generated O_3_/OH^·^ mixture activates PET surfaces creating peroxide and hydro peroxide groups^25–27^.
2. *Polymerization/grafting from* Three freshly ozonized PET fabrics were quickly placed in 60 mL degassed aqueous NaSS solution (0.7 mol L^−1^) under argon atmosphere at 70 °C under stirring for 1 h for the polymerization grafting-from reaction^10,11,24^. Peroxide groups created by the ozonation step were decomposed by 70 °C heating to generate radicals on the fiber surface from which PNaSS polymerization can initiate and propagate. These polymer functionalized samples were then extensively washed in distilled water for at least 48 h then freeze-dried under vacuum. PNaSS grafting of PET samples without SFR—i.e. PET fabric pieces without solvent extraction but with ozonation—was performed under the same conditions as a first control. Non-functionalized PET fabrics undergoing only SFR (no ozonation) were also used as a second control.

### Quantification of PNaSS polymer grafting on PET surfaces by toluidine blue (TB) colorimetric assay

PNaSS grafting rate (*GR*), that is, the amount of grafted sulfonate groups per mass of PET surface, on solvent-extracted PET samples was determined. Toluidine blue (TB) dye-depletion assay was used for quantitative analysis of sulfonate groups present on grafted PET surfaces since one molecule of TB quantitatively complexes one sulfonate group. This method has been previously described in detail^11,24^.

Briefly, PNaSS-grafted PET fabrics were cut into pieces (1 cm × 1 cm). Each PET fabric sample was incubated in 5 mL of 0.5 µmol L^−1^ TB solution (pH 10) for 6 h at 30 °C to achieve equilibrium complexation of TB/sulfonate groups (n = 3). TB-dyed fabric was 3-times washed with 5 mL of sodium hydroxide (1 µmol L^−1^ aqueous solution) for 5 min, then incubated in 10 mL aqueous acid acetic solution (50%, v/v) for quantitative decomplexation. The resulting TB concentration of the TB solution removed from PNaSS-grafted PET fabric was determined by UV–*vis* absorption measurement at 636 nm considering the TB concentration calibration curve. PNaSS *GR* (µmol g^−1^) was calculated as the following:

$$ GR = \frac{A \times V}{{\varepsilon \times L \times m}} $$

Given A as the measured optical absorption value of the TB decomplexation solution at 636 nm, V for the acid acetic volume (here 10 mL), ε for the molar extinction coefficient of TB determined from fresh TB solution and the optical density calibration curve (47,000–50,000 L mol^−1^ cm^−1^)^10,24^, L is path length (1 cm), and m is sample mass (g).

Nonspecific TB complexation in the case of aged or oxidized surfaces could be a limitation of this method; therefore, the reference TB value was that for TB binding to non-grafted PET fabric. Three samples were used for each analyzed surface.

### FTIR surface characterization

Attenuated Total Reflection-Fourier Transformation Infrared spectroscopy (ATR-FTIR) (Perkin Elmer Spectrum Two, PerkinElmer) with single reflection diamond crystal ATR cell (128 scans, 4000–400 cm^−1^ range, and 2 cm^−1^ resolution) was performed at room temperature (ambient atmosphere). Chemical characterization of SF oils and PET fabrics before and after the different treatment steps (i.e., SF as supplied, SF solvent removal, ozonation, PNaSS grafting) was performed and analyzed using Spectrum-10 software. The ATR crystal was extensively cleaned by ethanol and a blank scan in air was conducted before each experiment.

- A bundle of PET fibers was placed and pressed onto the crystal for analysis (n = 5)
- SFSB and SF-Ext oil were dropped directly onto the clean crystal for analysis (n = 5). In this case, DE or n-hexane or THF were used to clean the crystal and a new ATR reference spectrum in air was then collected.

### Analysis of fiber surface morphology and surface nano-mechanical properties

Surface morphology of PET fibers and fabrics samples was observed by Scanning Electron Microscopy (SEM) (TM3000, Hitachi-HighTech) and by atomic force microscopy (AFM) (Multimode-8, Brucker) at room temperature. In addition, the AFM apparatus is equipped with Peakforce-Quantitative Nano Mechanical (Peakforce-QNM) capability, a tapping mode integrated into the AFM system that allows (1) high resolution imaging, (2) resolving differences in surface mechanical properties, (3) quantifying mechanical properties by mapping on the nanometric scale the height, stiffness, elasticity and adhesion from surface force curves. Scanning was carried out in air with a silicon tip (triangular cantilever and rotated symmetric tip), on silicon nitride cantilever with reflective aluminum. Probe parameters include single probe cantilever with frequency f_0_ = 70 kHz and spring constant k = 0.4 N/m. Calibrating deflection sensitivity was performed for each experiment. The tip radius after absolute calibration is 1.78 nm. NanoScope Analysis 1.5 software was used. Measurement of Young’s modulus from the different fiber surfaces used the Force Volume program. Calibrating deflection was performed for each experiment. Experiments were performed in scan sizes 1.5 μm × 1.5 μm, at 3 different locations on 3 different PET fiber samples (under identical conditions), scan rate 1 Hz, 256 points/line. Atomic J software was used for calculating the Young’s surface modulus.

### Differential scanning calorimetric analysis

Differential scanning calorimetric (DSC) analysis was applied to measure the thermal transitions of PET fabric samples, and PET virgin film as control, before and after treatments using a DSC 8000 calorimeter (Perkin Elmer, Waltham, USA). Scanning was carried out under nitrogen atmosphere (N_2_ 20 mL/min, 2 bar) from 0 to 300 °C with a heating rate of 10 °C min^−1^, and results collected from the first scan (n = 3). Changes in observable thermal transitions of PET fabric samples as a function of sample age, degradation, oxidation and grafting reactions were analyzed, in particular, by variations in the PET melting enthalpy. The degree of crystallinity (X_c_) was calculated using the following equation:

$$ X_{c} = \frac{{\Delta H_{m} }}{{\Delta H_{m0} }} \times 100 $$

where ∆H_m_ is the melting enthalpy of the evaluated sample and ∆H_m0_ the theoretical 100% crystalline PET melting enthalpy (140.1 J g^−1^)^28^.

### Statistical analysis

Statistical analysis was performed using PAST v.4 software. Data compared for Young’s modulus used ANOVA and Tukey’s Pairwise analysis.

## Results

### Spin-finish oil degradation

**Figure 1:**
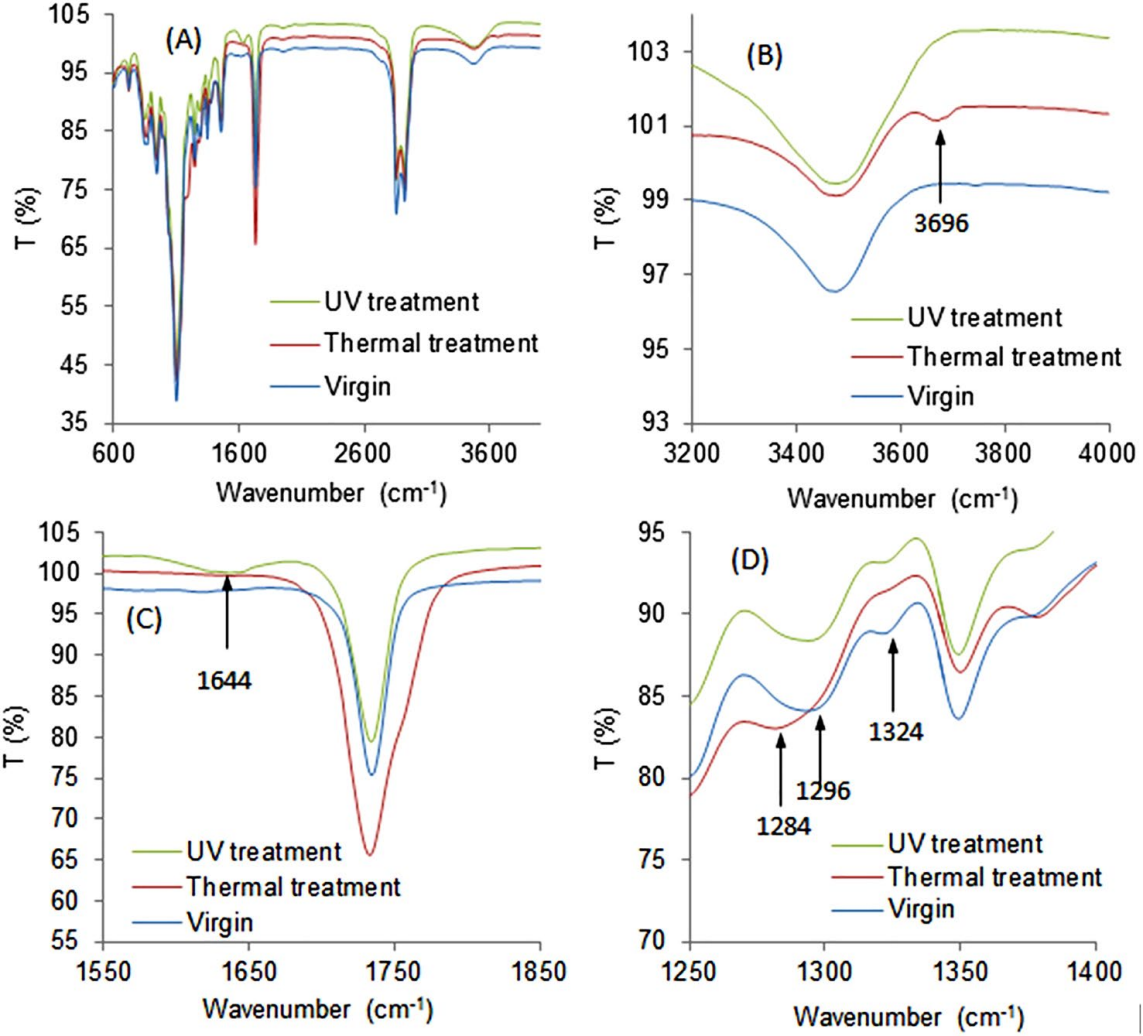
Infrared spectra of virgin (control), UV exposed (UV treatment) and heated (thermal treatment) SFSB oils. (**A**) Full FTIR of original and treated oils. (**B**) Resolved spectral region from 3200 to 4000 cm^−1^. (**C**) Resolved spectral region from 1550 to 1850 cm^−1^. (**D**) Resolved spectral region from1250 to 1400 cm^−1^. UV treatment was irradiation (254 nm); thermal treatment at 100 °C for 10 days, both under ambient atmospheric conditions.

**Figure 2:**
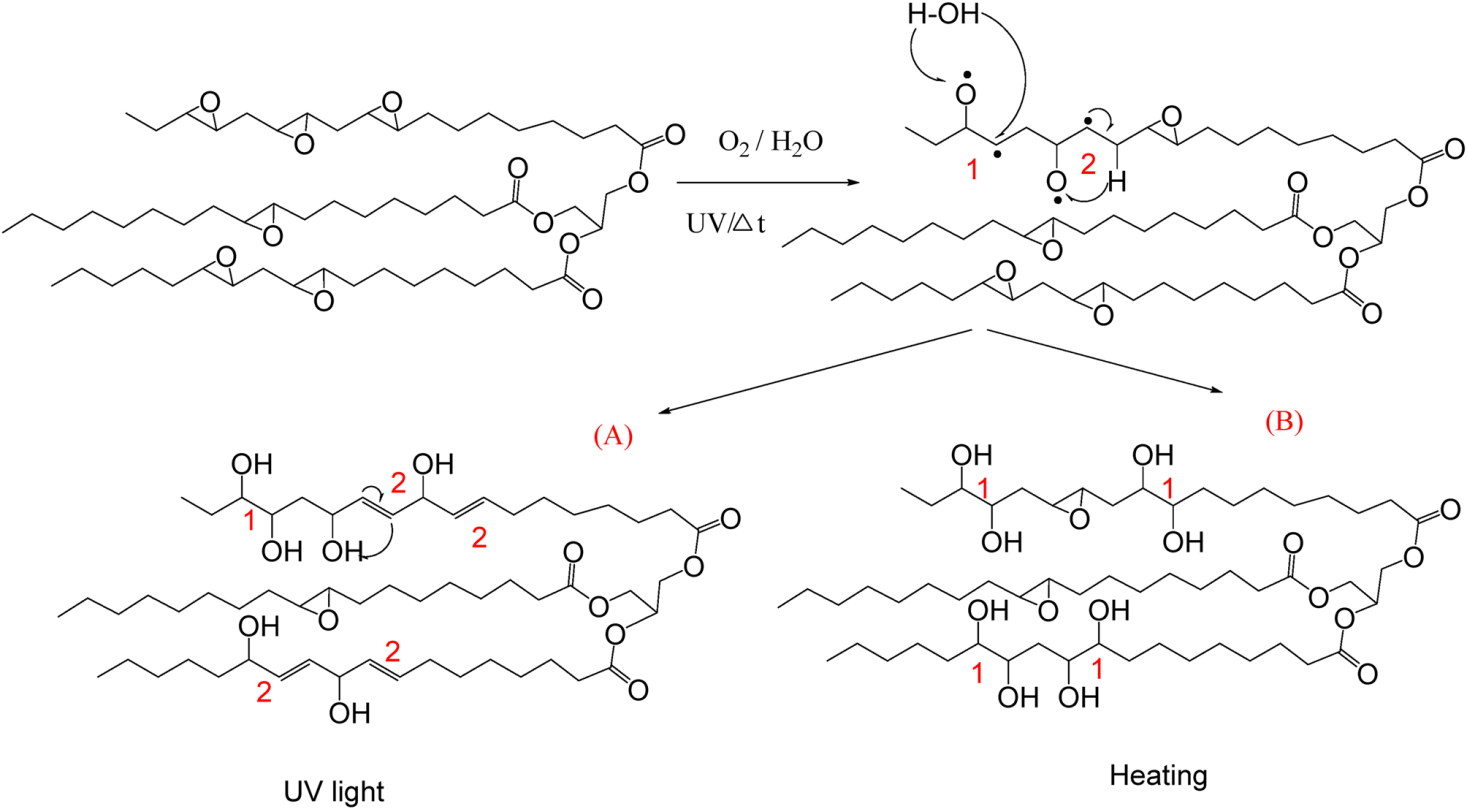
Scheme for modifications of epoxide soybean spin finish (SFSB) oil samples under (**A**) UV light irradiation (254 nm) and (**B**) heating (100 °C) for 10 days in air.

The ATR-FTIR spectra of virgin spin finish epoxidized soybean oil (SFSB)^29,30^ before and after accelerated degradation/aging by UV radiation (254 nm) or heating at 100 °C in air, are presented in Fig. 1. Spectral analysis provided evidence for the degradation mechanism of the extracted SF oil from PET fabrics. As shown in Fig. 1, UV-irradiation induced chemical modification to the SFSB oil illustrated by the appearance of a new vibrational band at 1644 cm^−1^ attributed to C=C vibration (see Fig. 1C), whilst thermal treatment led to the appearance of a sharp band at 3696 cm^−1^ attributed to free O–H stretching (see Fig. 1B). UV irradiation creates radicals that yield epoxy ring opening, leading to new C(OH)=C bonds (scheme Fig. 2). This correlates with the appearance of the new C=C band at 1644 cm^−1^ and the shoulder for the C(OH) at 3006 cm^−1^. Thermal effects are evidenced by generated OH groups’ sharp band at 3696 cm^−1^, attributed either to fatty acid oxidation or epoxide ring opening (scheme Fig. 2)^31^. SFSB oil under thermal treatment reduced the acyl chain R_1_–C(R_2_)=O vibrational band intensity at 1324 cm^−1^ (see Fig. 1D)^32,33^. Additionally, the C-O stretching vibration shifts from 1296 to 1284 cm^−1^ under thermal treatment (Fig. 1D), indicating that heating in air increases C–OH group formation^34,35^ and hydrogen bond formation between the molecules. On the other hand, both thermal and UV treatments intensified the SF-oil degradation by chain scission at the ester position and epoxy ring opening (decrease in 874 cm^−1^ peak) to form –OH and C=C chemistry^31,36^.

To summarize, two types of SFSB epoxy ring-opening reactions are observed (see scheme Fig. 2): reaction (1) heating leading to hydroxyl group generation, and reaction (2) UV treatment leading to de-hydroxylation reactions and resulting in alkene C=C double bond formation.

### Spin finish removal and surface analysis of long-term stored PET fabrics

#### Spin finish removal process

**Figure 3:**
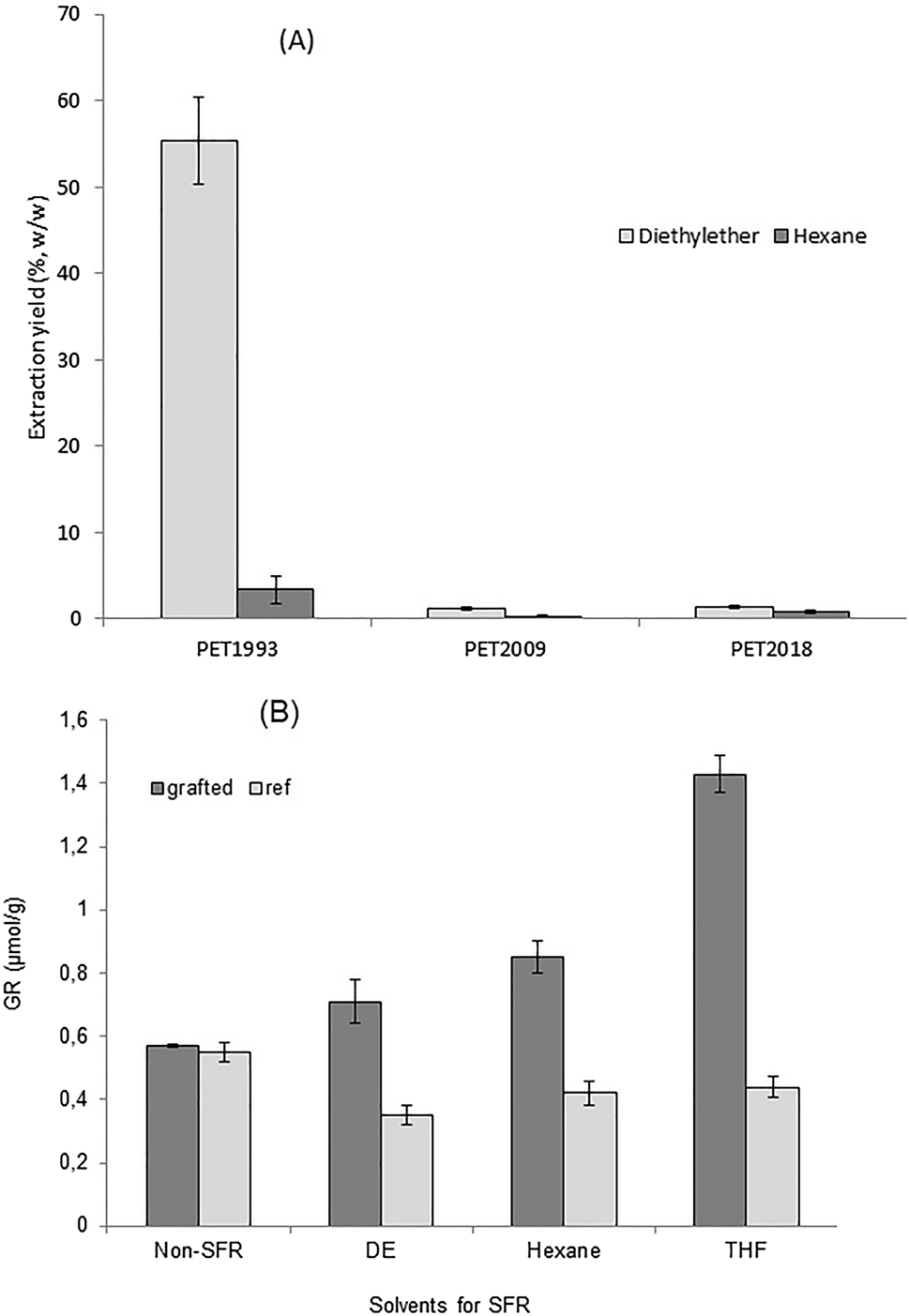
(**A**) SF oil extraction yields, *E*, from PET ligament fabrics of different storage times (i.e., from 1993, 2009 and 2018) using diethyl ether (DE) and n-hexane. (**B**) SFR solvent effects on the PNaSS GR on PET_2009_ ligament (Ref: virgin PET fabric but not PNaSS grafted); n = 3, *p* < 0.05.

Spin finish removal (SFR) of PET fabrics exploited Soxhlet extraction using three solvents selected for their ability to strongly solubilize SFSB oil and efficiently extract oil from the fabric while not affecting PET. Extraction from the selected solvents, n-hexane, DE and THF, and prior to PNaSS grafting were investigated (see Fig. 3). Solvents have generally weak polarities varying in the same direction as their dielectric constants, i.e. decreasing from THF to DE to n-hexane: ε_THF_ = 7.5, ε_DE_ = 4.3 and ε_n-hexane_ = 1.8.

Except for 26-year stored fibers (i.e., PET_1993_) in DE, the oil extraction yield, *E*, ranged from 1.2 to 1.4% in both n-hexane and DE (Fig. 3A). The extracted DE solution was evaporated to obtain the extracted oil from PET fabrics for FTIR analysis. N-hexane (boiling point 68 °C) and THF (boiling point 66 °C) were used as alternative extraction solvents to investigate chemical changes in spin finish oil (e.g., changing solubility from degradation, oxidation) from oil-coated PET fabrics ligament samples. However, n-hexane is reported to have limited extraction efficiency for soybean oil derivatives^37^, and THF could damage PET^38^.

Spin finish PET fabrics studied here are divided into two types: (a) long-term stored PET fabrics produced in 1993 (PET_1993_) that remained in contact with SF oil for 26 years before SFR; and (b) mid-to-short term stored PET fabrics produced in 2009 (PET_2009_) and 2018 (PET_2018_), remaining ten and two years, respectively, in contact with SF oil before SFR. All fabrics were stored in the same non-controlled ambient storage environment, and temperature maintained near 20 °C.

However, SF oil Soxhlet extraction efficiency was strongly modified by storage time duration between SF PET treatment and SFR: the longer the PET storage, the less effective the SFR extraction. Results expressed in % of extracted SF oil after 6 h of solvent reflux (see Fig. 3A) showed that extraction yields of more recent PET sheet fabrics produced in 2018 reached 1.4% and 0.87% (w/w) using DE and n-hexane, respectively. By comparing the 1.4% extraction yield of the most recent PET (PET_2018_) fabric with the initial SF content, the extraction conditions and yields are seemingly optimized. However, PET fabrics produced in 2009 exhibited a 15% and 80% decrease in extraction yield for DE and for n-hexane, respectively (i.e., to 1.2% and 0.3%, respectively). In addition, an unexpected, excessive amount of extracted oil was obtained from ~ 26-year old PET fabrics produced in 1993 (56%, w/w) from the unidentified SF oil chemistry applied at that time (Fig. 3A).

To investigate deleterious effects of these different storage times on subsequent PET PNaSS GR, solvents DE, n-hexane or THF were used for SF oil removal before performing PNaSS grafting on the two aged fabrics, PET_2009_ and PET_2018_ (see Fig. 3B). Results presented in Fig. 3B showed that:

- For PET_2018_, the obtained PNaSS GR was on the order of those already described by Ciobanu et al. (i.e., from 1 to 3 μmol/g)^10^.
- PET_2009_ showed a decreased obtained PNaSS GR related to the reduced SFR efficiency and oil extraction yield from extended storage.

THF extraction produced the highest effective SF oil removal compared to DE and n-hexane. This directly improves PNaSS GR for PET_2009_ which reached the GR value of most recent PET_2018_ fabrics, whereas DE and n-hexane extractions reduced the PET_2009_ GR.

#### Spin finish oil extracted from PET products

**Figure 4:**
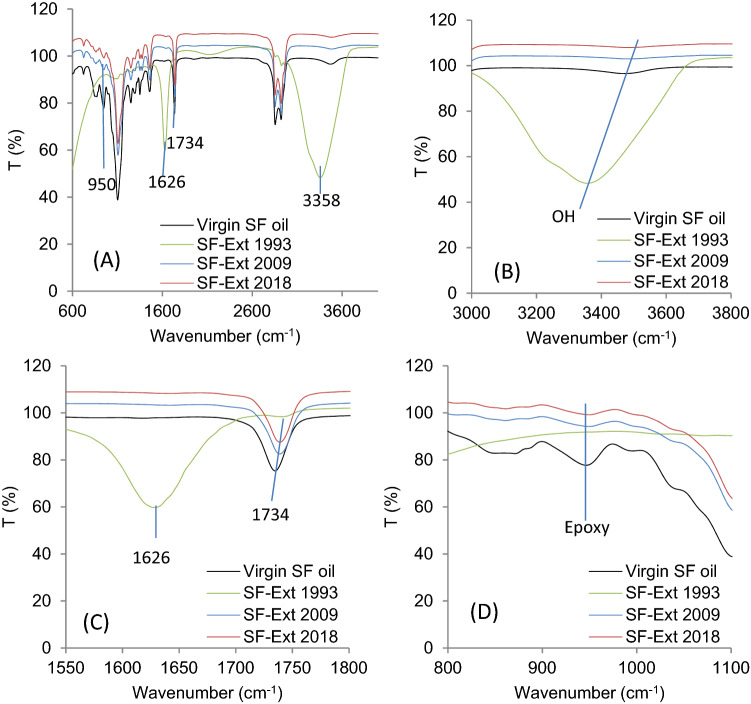
IR spectra of Soxhlet extracted oils from stored PET fabrics PET_1993, 2009, 2018_ compared to virgin SF oil (SFSB): (**A**) Overall spectra of spin-finish (SF) oil, extracted oil from PET (_1993, 2009, 2018_) samples, respectively, (**B**) regional spectra from 3800–3000 cm^−1^, (**C**) regional spectra from 1800 to 1550 cm^−1^, (**D**) regional spectra from 1100 to 800 cm^−1^.

Finishing oil degradation was analyzed in solvent extracts from PET samples by FTIR spectroscopy. IR spectra of the different oil extraction processes from PET_1993,_ PET_2009_ and PET_2018_ are displayed in Fig. 4. Results show significant differences between spectra of virgin SF oil (non-degraded SFSB) and oil solvent-extract spectra from PET_1993,_ PET_2009_ and PET_2018_ fabric samples. PET_1993_ oil extracted from 26-year storage showed (1) a strong and broad OH peak at 3358 cm^−1^ (Fig. 4B); (2) a 1626 cm^−1^ C=C stretching bond vibrational intensity overwhelming that for C=O at 1734 cm^−1^ (Fig. 4C) that declines^39^. The emerging C=C bond vibration could be generated by UV exposure of the oil through epoxy ring opening^40^ (see scheme in Fig. 2). The epoxy vibration identified at 950 cm^−1^ dissappeared for extracted oil from PET_1993_ and decreased for PET_2018 and 2009_ oil samples compared to virgin SF-oil (see Fig. 4D).

#### Degraded spin finish oil interaction with PET fiber surfaces

**Figure 5:**
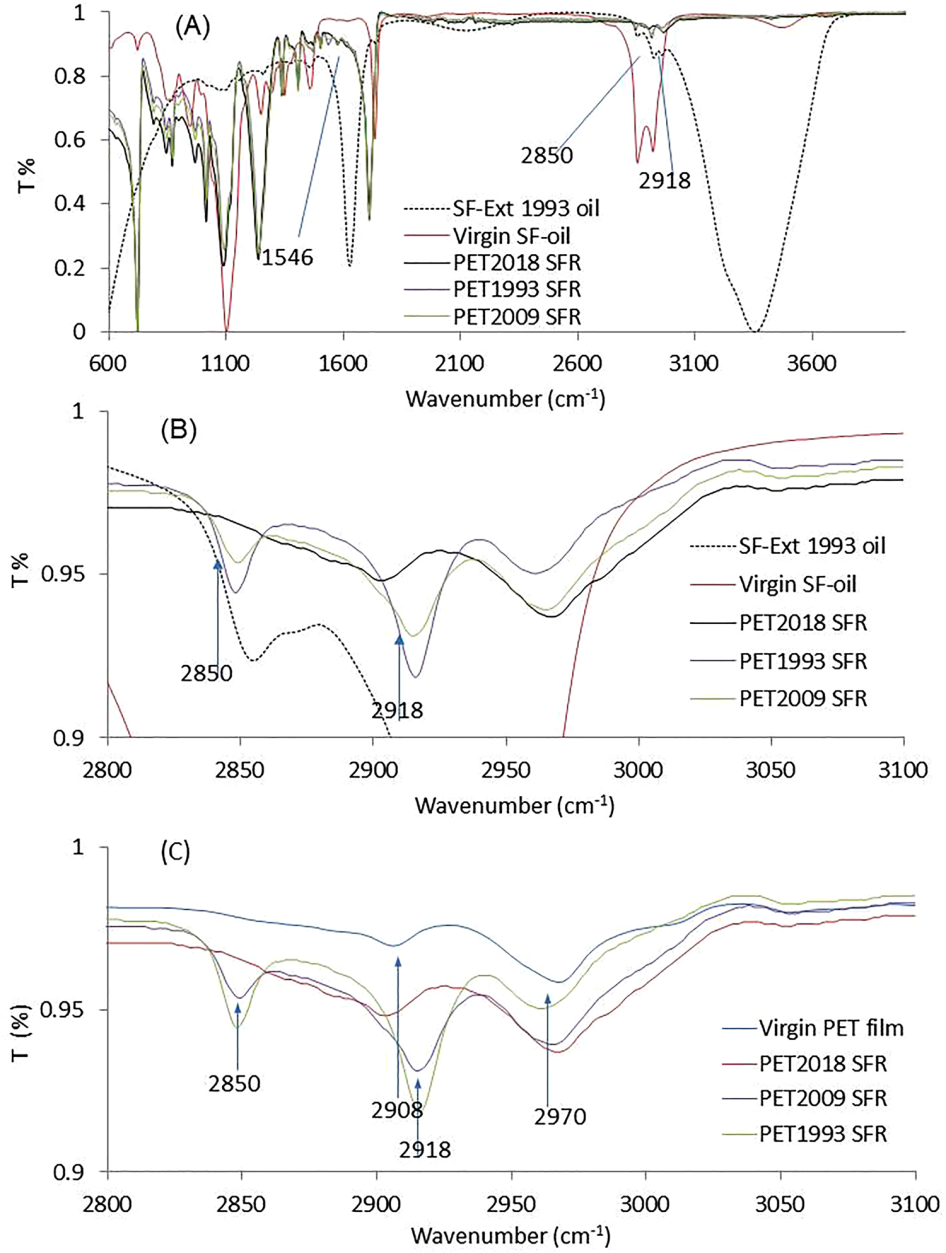
IR spectra of SFR-extracted PET fabrics (from 2018, 2009, and 1993) compared to virgin SF oil (non-degraded) and SF-Ext oil (degraded oil extracted from PET_1993_ fiber): (**A**) Overall spectra of spin-finish oil (virgin SF oil versus degraded, extracted oil from PET_1993_) and spin finish oil-removed PET fabrics, (**B**) regional spectra from A, shown from 2800 to 3100 cm^−1^, and (**C**) spectrum of virgin PET film as a reference for spectral changes from spin finish removal from PET ligaments (regional spectra from 2800 to 3100 cm^−1^).

To explain the observed reduced PNaSS *GR* on mid-to-long storage time PET samples, we analyzed the reduced efficiency of SFR and the SF oil remaining on PET_2009, and 1993_ samples. FTIR spectra of these sample extracted PET fabrics, presented in Fig. 5A, show peaks at 2850 cm^−1^ and 2918 cm^−1^_,_ characteristic of asymmetric C–H stretching (2918 cm^−1^) and symmetric C–H stretching (2850 cm^−1^) vibrations from methylene groups of both non-degraded virgin SF-oil and degraded SF-Ext (i.e., fiber extracted) oil (Fig. 5B,C). Indeed, the appearance of the 2850 cm^−1^ peak on samples PET_2009_ and PET_1993_ corresponding to 2 characteristic peaks of virgin SF-oil, supports presence of residual organic molecules on PET fiber surfaces even after SFR for these aged samples. This 2850 cm^−1^ signal was not evidenced on either PET_2018_ or reference PET film. In addition, SF-Ext 1993 oil (i.e., SF oil extracted from PET_1993_) also showed the 2850 cm^−1^ peak. In other words, residual SF-oil could remain on PET_1993_ and PET_2009_ fiber surfaces after the solvent extraction process (SFR). In contrast, the newer PET_2018_ surface showed a surface devoid of SF oil after the SFR process.

#### SEM analysis

**Figure 6:**
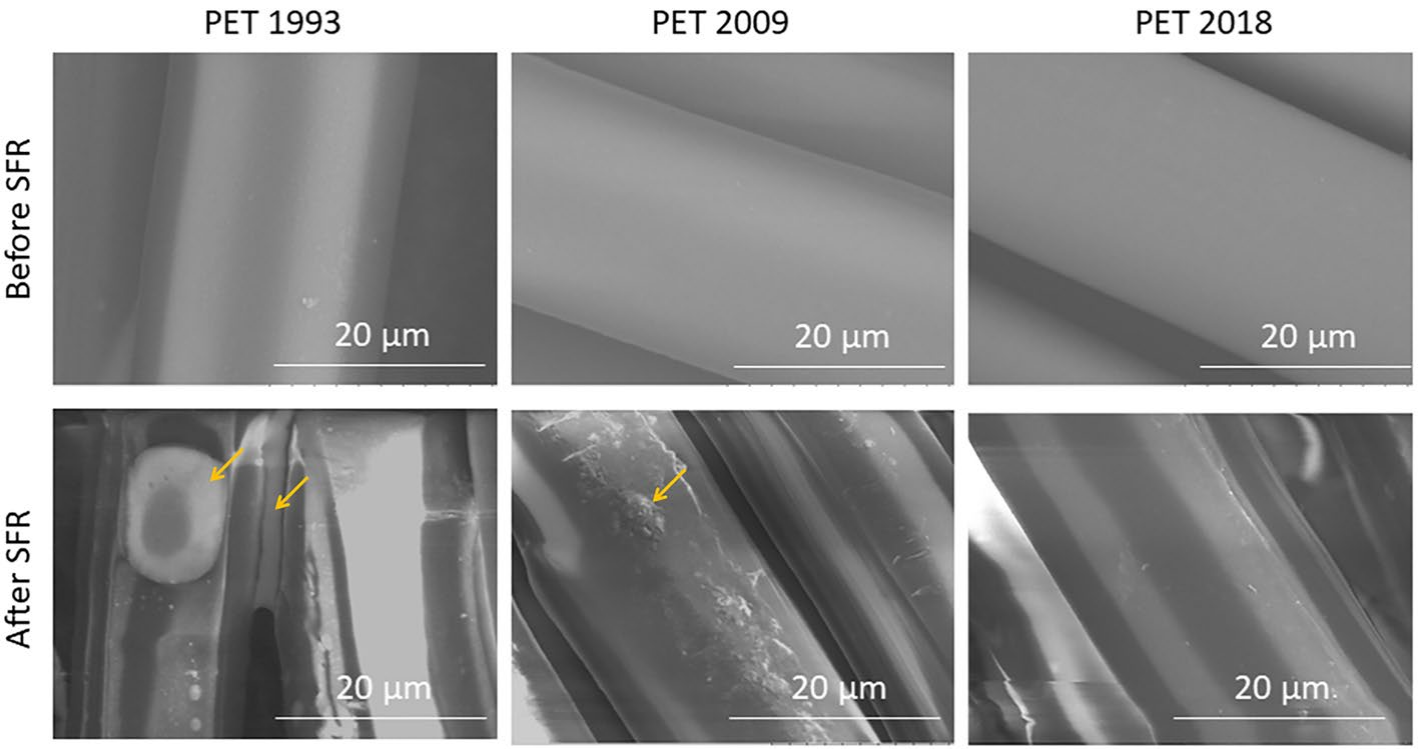
SEM images of PET fiber surfaces before and after Spin Finish Removal (SFR) under diethyl ether (DE) extraction; Yellow arrows indicate oil droplets or contaminants remaining on PET surfaces after SFR. Scale = 20 µm.

The observed chemical alterations of PET spin finish oil with storage prompted further analysis of the extracted PET fiber surface structure using scanning electron microscopy (SEM). SEM images of the different aged PET fabrics PET_1993, 2009, 2018_ before and after solvent SFR are presented in Fig. 6.

SEM results showed: (1) that PET fiber surfaces of all ages were smooth before SFR, (2) remaining oil or surface contaminants (Figure A.5, supplementary information) after SFR when using DE are evident as round or spread droplets on PET_1993_ and PET_2009_ (yellow arrows*,* Fig. 6), whereas no such evidence is detected on PET_2018_.

Remaining oil or surface contaminants after SFR of these PET fabrics from the two longer storage times is attributed to SF oil aging/oxidation/degradation, subsequent reduction in SF oil solubility and resulting limited extraction in DE solvent with aging. This also confirms strong PET surface interactions with adsorbed, degraded finish oil. Longer storage indicates degradation of the PET interface and/or changes in the finishing oil over time.

#### DSC thermal analysis

**Figure 7:**
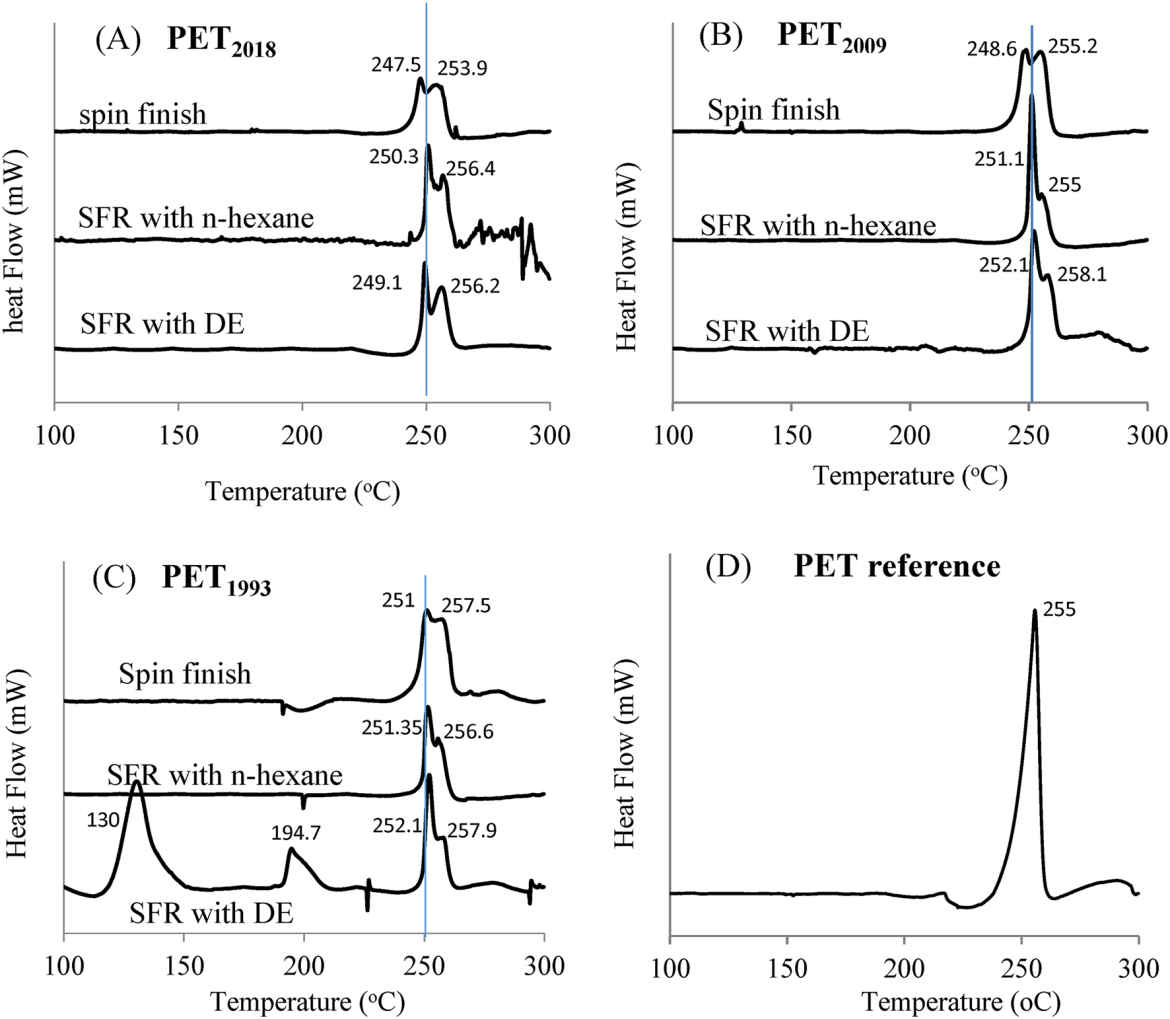
DSC thermal analysis first scan for PET fibers sourced from 1993, 2009, and 2018 before and after spin finish removal (SFR) with n-hexane and diethyl ether (DE): (**A**) PET_2018_, (**B**) PET_2009_, (**C**) PET_1993_, and (**D**) PET reference film; (n = 3).

**Figure 8:**
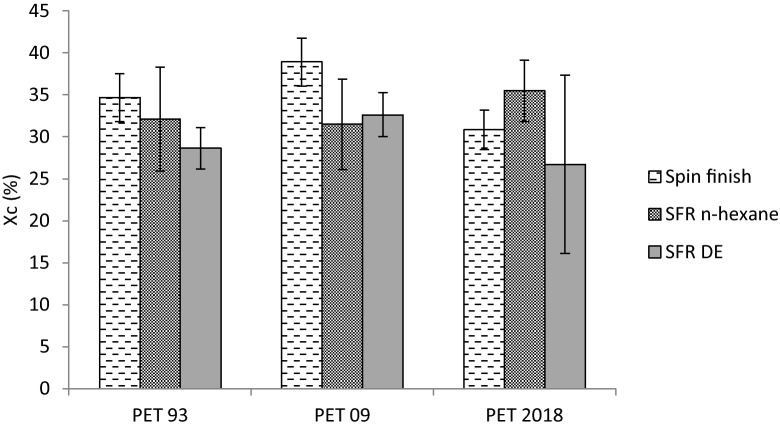
PET crystalline fraction (X_c_) determined by DSC scans for PET fiber samples stored since 1993, 2009 and 2018, both before and after SFR using n-hexane and DE.

PET fabrics from different storage times (PET_1993_, PET_2009_, PET_2018_) and a PET reference film used as control were analyzed by Differential Scanning Calorimetry (DSC). DSC scans performed before and after SFR for fabrics are presented in Figs. 7 and 8, with melting temperatures (T_m_, Fig. 7) and calculated fractional crystallinity (X_c_, Fig. 8) compared to that of PET control (virgin semi-crystalline PET film). Analysis and comparison of the different DSC thermal scans from Fig. 7 showed:

1. Differences in PET melting temperatures (T_m_) between PET control (single peak at 255 °C) and PET fabrics/fibers (two melt peaks—first ~ 250 °C and second between 255–258 °C, Fig. 7). These differences can be explained by different PET sample processing—flat extrusion for the PET control, and extrusion plus fiber stretching followed by unknown but specific heat treatment for the PET fibers used to weave the fabrics. Extrusion and heat treatment are proposed to strongly modify resulting polymer crystalline structures of the thermoplastic polymer PET; in the case of fiber extrusion and extension, application of uniaxial tensile stress increases again the orientation of macromolecular chains along the fiber, leading to partial crystallization and increases to PET T_m_. The appearance of double T_m_ peaks has been attributed to different polymer crystal lamellae thickness: Kong and Hay demonstrated that the secondary melt peak was produced by polymer re-crystallization in the thickest lamella^41^.
2. DSC scans of spin finish PET fabrics—i.e. without SFR—are very similar, independent of their storage time, indicating that spin finish oil maintains the integrity of the PET fibers and fabrics (Fig. 7).
3. The SFR effect on the DSC profile depended strongly on solvent extraction efficiency (Fig. 7):
  N-hexane is less efficient solvent for the SFR process, leading to similar DSC profiles for the three samples because the solvent can only extract the superficial layer of oil on fabrics, leaving residual oil on the fiber surface (see also Figs. 3, 5);
  DE was a more efficient solvent for SFR (see Figs. 3, 5). DE extracted oil from PET_2018_ and _2009_ in a similar way, giving identical DSC scans, whereas for PET_1993_, DE produced different results. DSC was not sufficiently sensitive to directly demonstrate PET surface degradation, but was useful to discern differences in solvent extraction efficiencies that indirectly reflect that possibility. We propose that DE dissolved the SF oil on PET_1993_, plus an additional oil/degraded PET layer, leaving a damaged PET surface, with DSC peaks attributed to possible remnant PET oligomers (two first peaks) and the actual bulk PET melting peak (255 °C)^42^.

As seen in Fig. 8, no statistically significant variations in PET crystalline fractions (X_c_) are observed after SFR, using either n-hexane or DE. Additionally, as different commercial PET fibers samples were fabricated in different batches, absolute values of PET crystallinity determined by DSC cannot be directly compared.

Furthermore, DSC scans were used to estimate physical aging of PET fiber samples. This phenomenon can be verified when, during the manufacture, amorphous or semi-crystalline polymers are cooled quickly bellow the glass transition temperature (T_g_) resulting in a non-equilibrium (quenched) polymer state. Once solidified, polymers can undergo slow solid-state structural relaxation towards thermodynamic equilibrium. These structural adaptations induce stabilizing mechanical and structural property modifications, and their T_g_ then increases. Unfortunately, heating inherent to SFR fiber extraction could alter this thermodynamic process for PET samples after SFR treatment. Nonetheless, observed changes in T_g_ over storage (i.e., in PET_1993_, PET_2009_ and PET_2018_) were evaluated and results shown in Table 1 confirming PET fiber T_g_ changes over years.

**Table 1:** Glass transition temperature (T_g_) of PET ligament fibers after SFR treatment.

Sample	PET 2018	PET 2009	PET 1993
T_g_ (°C)	68.2 ± 7.8	95.0 ± 12.6	89.5 ± 10.3

### Topography and surface mechanical parameters of long-term stored PET_1993 and 2009_ fabrics before and after PNaSS grafted surface functionalization

#### Fiber topography analysis before and after grafting-from surface functionalization

**Figure 9:**
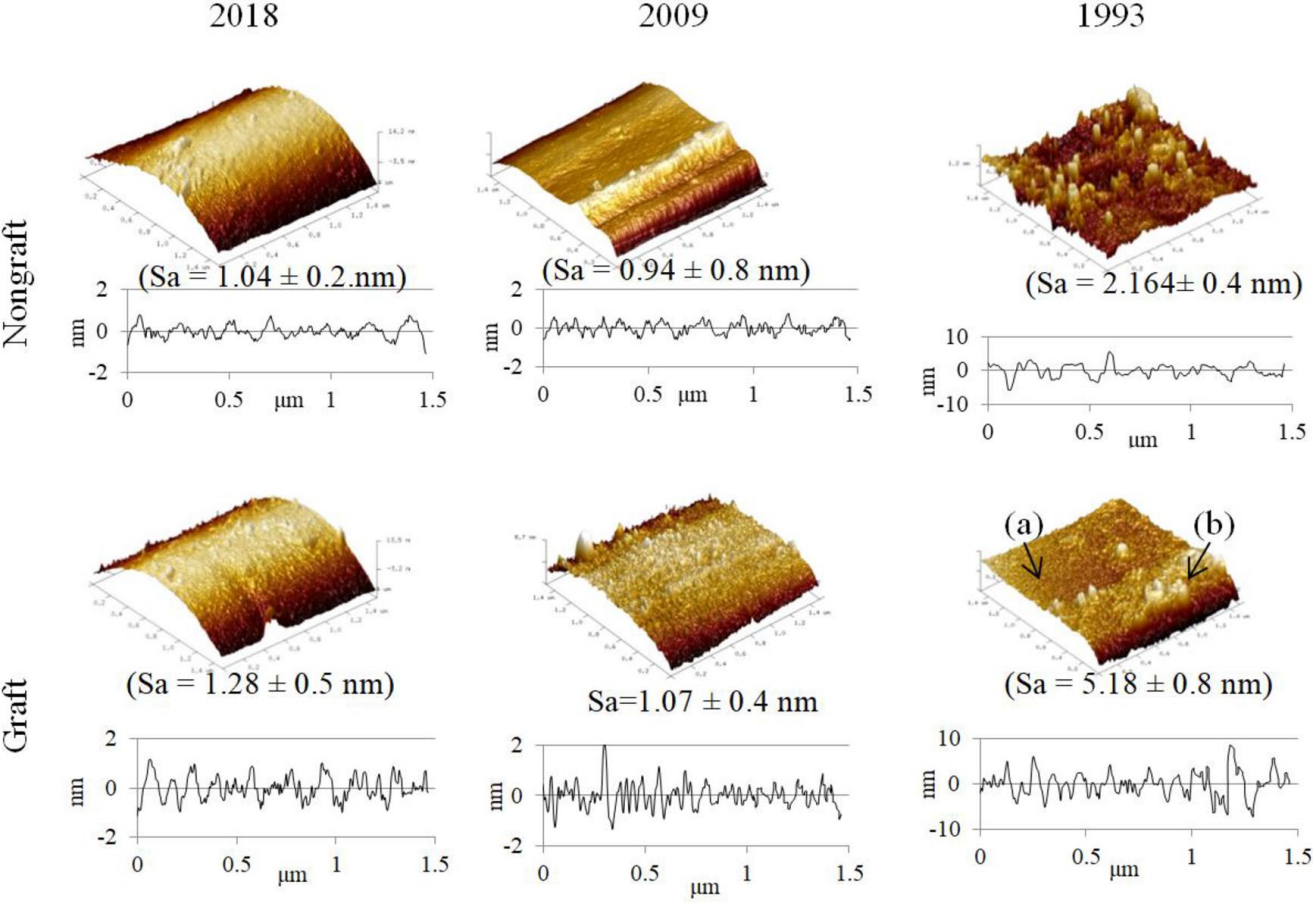
AFM imaging of PET fiber topography and roughness for fiber surfaces treated by SFR and PNaSS grafting protocols after different aging times (shown above each column); Grafting conditions: PET samples subject to SFR and then PNaSS functionalization protocols of ozonation for 10 min at 30 °C and grafting for 1 h at 45 °C. AFM scan area: 1.5 μm × 1.5 μm (0.5 Hz) in air at 25 °C (n = 3).

AFM analyses of PET fabrics samples before and after PNaSS functionalization are presented in Fig. 9. The method seeks to compare effects of surface treatments including spin-finish removal (SFR) and presence or absence of PNaSS grafting on resulting PET surface topography and surface mechanics from samples of different storage durations. Similar to SEM imaging (Fig. 6), finishing oil residues still present on PET surfaces after SFR by DE are detected by 3D topographical imaging of both non-grafted and PNaSS-grafted PET_1993_. AFM imaging allowed further demonstration that before PNaSS grafting, PET_1993_ fiber surfaces are still covered by oil or oil-swollen PET layers as reflected by surface roughness values (Sa) equaling 2.164 ± 0.4 nm. This Sa value was higher than that observed for both non-grafted PET_2009_ and PET_2018_ fibers, respectively (i.e., 1.04 ± 0.2 nm and 0.94 ± 0.8 nm, respectively). Additionally, after PNaSS grafting, surface roughness of PET_1993_ fibers increased to a Sa value of 5.18 ± 0.8 nm, much higher than that for non-grafted and other samples. In fact, the surface textures of PNaSS-grafted PET_1993_ are divided into basic two types: (a) smooth and (b) rough surfaces (Fig. 9 and supplementary Fig. A.3). This is explained by influences or differential effects of the PNaSS functionalization process in the presence or absence of finishing oil residue. Region (a) resulted from removing the finishing oil layer during the ozonation and PNaSS grafting, hindering peroxide generation and PET fiber surface etching, while residual oil layer in region (b) remained on the PET surface despite SFR, limiting polymer grafting and ozone etching (Fig. A.3).

In contrast, the entire surfaces of PET_2018_ and PET_2009_ were “cleaner” after SFR with quite low surface roughness, Sa = 1.04 ± 0.2 nm for PET_2018_ and 0.94 ± 0.8 nm for PET_2009_ (see also Fig. 6). A slight increase in surface roughness was recorded for PNaSS-grafted PET_2018_ and PET_2009_ fibers, with Sa values of 1.28 ± 0.5 nm and 1.07 ± 0.4 nm, respectively. This corresponds to the observed increased PNaSS GR (see Fig. 11) and demonstrates the efficiency of DE SFR treatment and resulting PNaSS grafting on these PET fabric samples after SF oil removal.

#### Peakforce quantitative nano-mechanical properties AFM analysis

**Figure 10:**
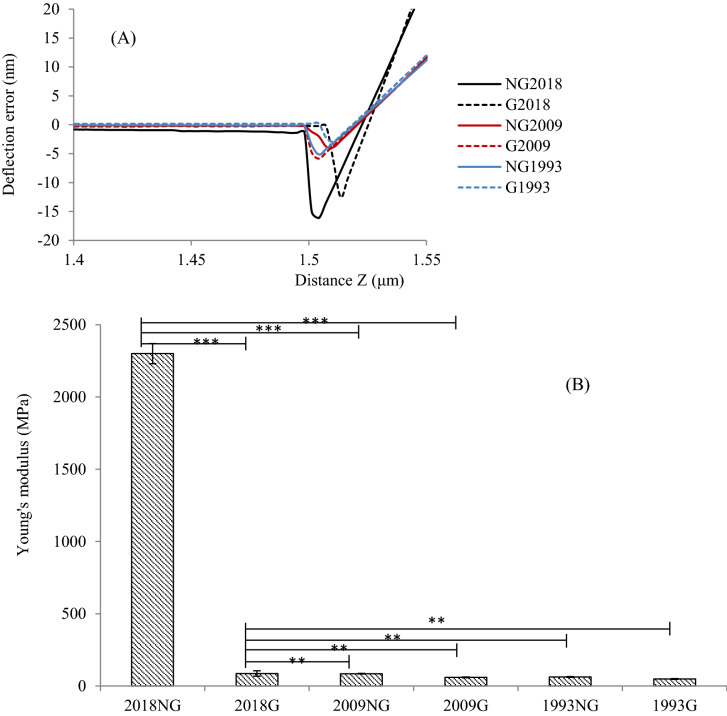
AFM-derived: (**A**) Force-distance curve and (**B**) Young’s surface modulus for PET2018, PET2009, and PET1993 before and after PNaSS grafting (NG: non-grafted and G: grafted). Measurement in air (25 °C), 256 points/line, n = 3, **p < 0.001, ***p < 0.05.

AFM force curves and Young’s modulus changes confirm surface property modification of PET fiber samples after the different storage, treatments and processes. AFM force curves were collected by the Force Volume program. Retracted force curves (Fig. 10A) on PET_2018_ surfaces for both virgin and non-functionalization are clearly higher than others. This is evidence for a harder surface of the newer PET fiber^43^. Likewise, low deflection error collected from PET_2009 and_ PET_1993_ fibers (Fig. 10A) is associated with interactions between cantilever tip and soft and wettable surfaces^43,44^. This demonstrates and supports further PET surface modification from the SF oil over time—either the oil itself changes and resulting PET surface interactions are physically and/or chemically changed, or the PET surface is changed, or both. Possibly, both polymer and finishing oil degradation results in PET chain scission on these two aged oil-coated PET samples.

PET surface Young’s modulus (E_γ_) was measured (Figs. 10B, A.4). Surface Young’s modulus of SFR non-grafted PET_2018_ was measured to be 2.30 ± 0.07 GPa (comparable to 2–4 GPa from literature)^2,44^ and much lower values obtained for non-grafted PET_2009_ with E_γ_ = 85 ± 2.19 MPa (Fig. 10B). Young’s surface modulus for non-grafted PET_1993_ decreased significantly with E_γ_ = 63 ± 3.09 MPa. After PNaSS grafting, E_γ_ of grafted PET_2018_ was substantially reduced to 86.92 ± 18.74 MPa, resulting from the new, lower modulus PNaSS layer combined with likely minor surface changes from PET functionalization processes^24^. Non-grafted PET_2009_ shows a lower Young’s surface modulus (~ 85 MPa), with further surface modulus reduction after polymer grafting, similar to that of PET_2018_. The surface modulus of non-grafted (~ 63 MPa) and grafted PET_1993_ (E_γ_ = 48.72 ± 2.53 MPa) fiber were both substantially below less-aged PET fibers, reflecting more drastic oil and PET fiber physical and chemical changes, and grafting changes to these oldest samples.

Such changes observed in Young’s surface modulus possibly correlate with PNaSS grafting efficiency (GR) on each PET sample. Indeed, the high PET_2018_ Young’s modulus value of E_γ_ = 2.3 GPa reflects an intact non-degraded PET surface. Grafting processes reduce the PET_2018_ surface modulus to 87 MPa (96% decrease). Substantial surface PET oxidation under ozonation promotes efficient PNaSS grafting from this PET surface. For PET_2009_, Young’s surface modulus of non-grafted fiber samples is similar to the grafted PET_2018_ fiber surface, attributed to PET fiber surface degradation by PET and SF oil oxidation during long-term storage, even though this PET fabric sample was covered by SF oil, and then further surface degradation from the polymer grafting protocol. The observed E_γ_ reduction of PET_2009_ from ungrafted 85 MPa to grafted 60 MPa (decrease of 29%) reflects these similar surface alteration phenomena as with PET_2018_. Overall, the E_γ_ for grafted PET_2009_, non-grafted PET_1993_, and grafted PET_1993_ were comparable (i.e., 48–63 MPa), reflecting roughly equivalent altered PET surface physical states for these fabrics after these aging and/or surface treatments.

While these results support limited degradation of PET_2009_ fibers from polymer grafting, the topography (Fig. 9) and Young’s surface modulus measurement (Fig. 10) on PET_2009_ support their usability.

### PNaSS grafting rate (GR) measurements

**Figure 11:**
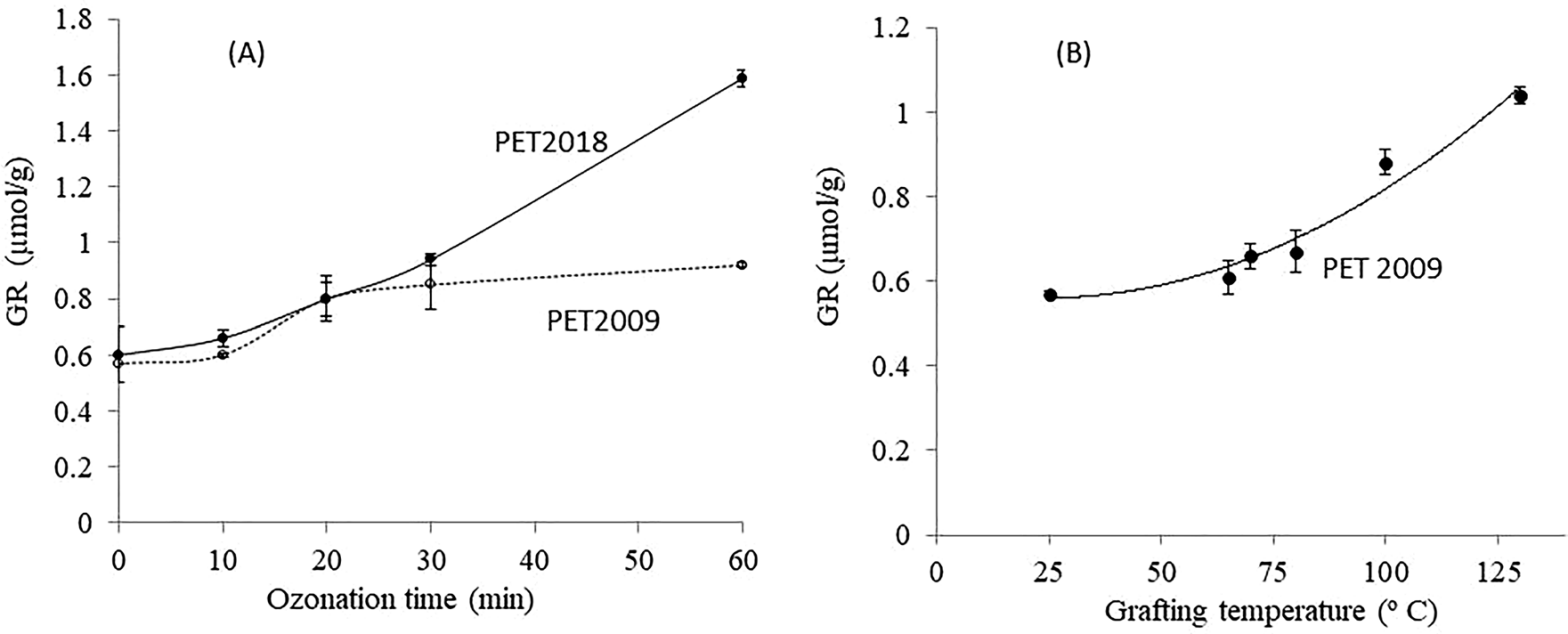
(**A**) Comparing functionalization of PET_2018_ and PET_2009_ (ozonation at 30 °C for various times from 10 to 60 min, grafting at 45 °C for 1 h) (n = 5). (**B**) Improving PNaSS *GR* of PET_2009_ fabrics by elevating PET grafting-from processing temperatures for 1 h at 25, 65, 70, 80, 100, 130 °C (n = 3) (ozonation 10 min at 30 °C); SFR used diethyl ether (DE).

Colorimetric Toluidine Blue (TB) dye binding was used to determine the PNaSS GR for PET_2009_ and PET_2018_ surfaces after solvent extraction with DE and then different ozonation times. GR results versus ozonation time, presented in Fig. 11A, show that for PET_2009_ fabrics (i.e., 10 year storage), GR saturates after 20 min of ozonation whereas GR of PET_2018_ increases gradually from 0.6 to 1.6 µmol g^−1^ h^−1^ with increasing ozonation time^10,24^. Nonetheless, the effects of different SF oil chemistry and resultant degradation and oxidation in storage, subsequent interactions with PET fiber surfaces and ambient water, and extraction efficiency in SFR based on these chemical transformations are all clearly involved in the final fiber ozonation functionalization efficiency and therefore the fiber *GR*.

To improve the *GR* for PET_2009_, a further study using increased fiber grafting temperature (Fig. 11B) shows that PNaSS *GR* could achieve about 1 μmol g^−1^, similar to the *GR* for PET_2018._

## Discussion

### Influence of ozonation exposure and spin-finish oil/PET interaction on PET surface reactivity during polymer grafting-from functionalization

#### Ozonation exposure

Functionalization of PET fibers/fabrics by ozonation and PNaSS grafting was performed on the different aged and extracted PET fabric surfaces. PNaSS *GR* for PET fabrics _1993, 2009 and 2018_ was measured using the TB colorimetric assay (Figs. 3B, 11). Only a fraction of radicals generated during this ozonation and thermal polymerization “grafting from” processes are presumed to actually participate in initiating NaSS polymerization and grafting from each PET surface. Indeed, each radical will not initiate a single PNaSS grafted chain due to known competition between polymerization from surface radicals and those initiated in solution, disproportionation effects, and radical quenching by other species in the system. Surface radicals may be “de-activated” to non-reactive hydroxyl groups (–OH) before encountering NaSS monomer molecules^1,25,26^. Therefore, PNaSS-grafted surfaces are functionalized by propagating PNaSS chains while also generating -OH surface groups from hydrolyzed peroxide. Increasing GR with increased ozonation time demonstrates that more peroxide groups are continually generated on PET_2018_ surfaces to provide radicals for initiating NaSS polymerization^10,24^ (Fig. 11). In contrast, PET_2009_ fiber surfaces do not generate radicals/–OH groups with increased ozonation time (Fig. 11), implying that PET_2009_ surfaces are degraded and chemically compromised before grafting functionalization.

#### Effect of oil/PET interaction after long-term storage on spin finish removal and functionalization

Sizing or spin finish (SF) treatment is widely used in the textile industry to improve fiber extrusion and handling for enabling further knitting or weaving of fabrics^17^. Nevertheless, before starting any additional fiber or fabric surface treatments—here, the required PNaSS grafting of medical PET implant fabrics for biocompatibility—ideally, total removal of the spin finish oil is required using SFR methods. Indeed, SF oil residue prevents polymer radical initiation and PET surface grafting; therefore, instead of initiating polymerization, radicals will induce oil degradation and prevent PNaSS-PET surface grafting to proceed as desired.

**Figure 12:**
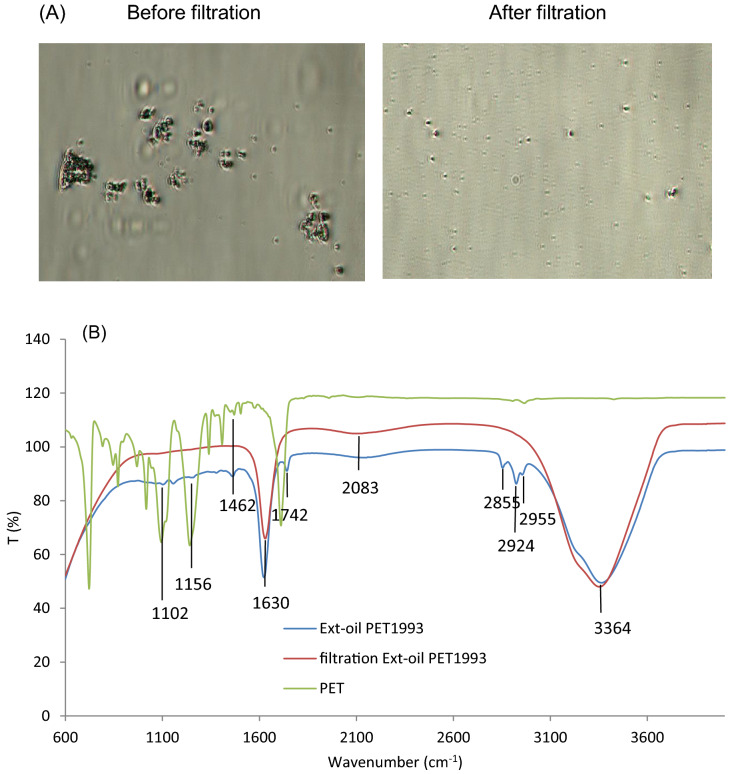
(**A**) Optical microscopy observation of PET-Ext oil from PET_1993_ before and after filtration showing particle presence in suspension. (**B**) FTIR spectra of extracted SF oil from PET_1993_ before and after filtration compared to PET spectra.

Degradation of SF oil on long-term stored PET fabrics was investigated. Combining the extraction yield (55.6% extracted oil collected from PET_1993_) (Fig. 3A) and FTIR results (Figs. 4, 5) provides clear evidence for formation of hydroxylated fatty acids from oil oxidation long term. In addition, extracted oil from PET_1993_ was observed by optical microscopy, revealing several solid particles in the oil that disappear after passage through a membrane filter (0.22 µm). The FTIR spectra of the oil before and after filtration (Fig. 12) reinforce the theory that these particles are indeed extracted PET oligomers, and the results support that, for long-term storage, oil degradation and interaction with the PET substrate can produce degradation by-products in both oil and PET materials^23^.

The solubility of finishing oil extracted into SFR non-polar solvents decreased in the following order: THF > diethyl ether > n-hexane (see supporting supplementary information Fig. A.2). This partly explains the significantly increased oil extraction yield for PET_1993_ where degraded finishing oil increased extracted oil product mass considerably (Fig. 3).

Additionally, spin finishing oil present after longer fiber storage times can degrade/modify PET surfaces as well as the SF oil as shown in Fig. 4. This changes the SFR result and might also lead to inhibited or limited ozonation activation steps on these surfaces. Aging and degradation are reported to induce a very low-to-none peroxide generation on PET_2009_ fabrics: PET surfaces were hydrolytically degraded, leading to chain scission and producing hydroxyl groups that prevent peroxide generation^1,27,41,45^. Particularly, due to aging, PET_1993_ fabrics no longer comprise virgin PET fibers but have become PET fibers highly impregnated at surfaces with degraded oil (Figs. 6, 9); the outer polymer surface layer of the PET fibers comprises a polymer/oil mixture (Fig. 5)^22,31,46^. SFR oil extracts from PET_1993_ are shown to contain degraded oil and particles attributed to degraded PET (Fig. 12), accounting for the excess extraction efficiency, *E*, seen only on this sample (Fig. 3A). Therefore, long-term storage of spin finish-treated PET fabrics or fibers compromises SFR, increases PET surface degradation, compromises oil protection, and reduces PNaSS GR.

These collective results indicate that without proper storage precautions, or under UV and/or oxygen/water exposure from ambient atmosphere, SF oil and PET surfaces could be chemically degraded over time as follows:

1. Radicals can be generated on PET macromolecular chains as described^27^, producing chain cleavage and oxidation chemistry as evidenced in altered surface FTIR/ATR spectra (Figs. 1, 2) and in Young’s surface modulus by AFM (Fig. 9);
2. Unsaturated hydrocarbon chain olefin bonds and epoxides in spin finish-derivatized soybean oil^39,40,47,48^ promote free radical generation and oil oxidation (Figs. 1, 2, A.1);
3. Radical and/or oxidative chemistry propagation to and cross-linking of oil-PET at the fiber interface can occur (Figs. 5, 6, 9)^31^.
4. Degraded oil is chemically distinct from virgin SF oil and can also interact with degraded PET surfaces (see Fig. 2)^1,27,41^ through new hydrogen bonds via oxidation by-products (see Fig. 6);
5. While virgin semi-crystalline PET is not highly water absorbing, water and oil can be absorbed onto/into the more polar oxidized PET/oil fiber surface, leading to swelling of oil layers and increased mass of extracted oil with possible entrained degraded PET material with more polar SFR solvents (Figs. 3, 4).

**Figure 13:**
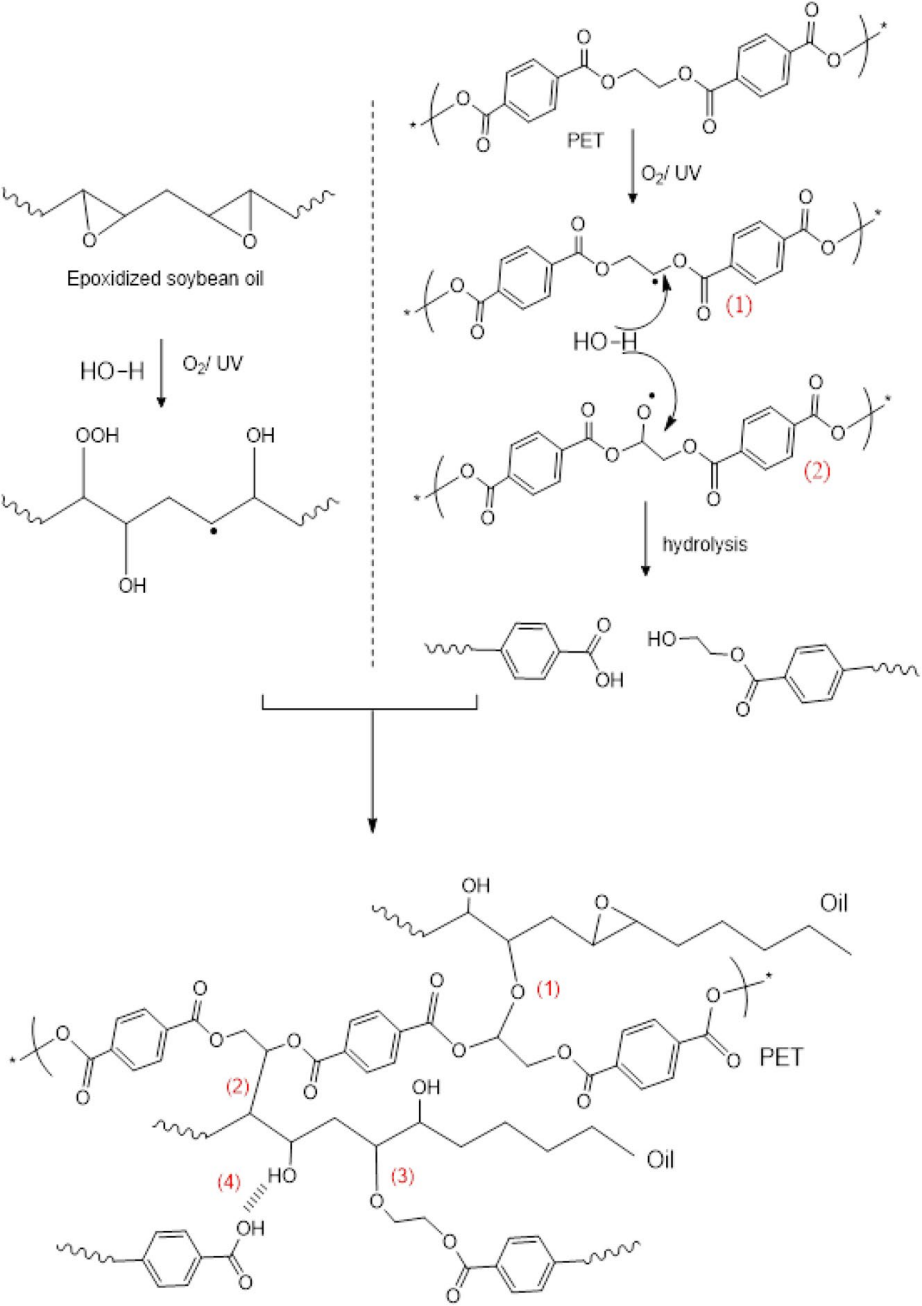
Schematic for UV/O_2_ effects on SF-oil and PET interactions: UV/O_2_, temperature, and humidity yields intermolecular crosslinking and increased hydrogen bonding between oxidized oil and PET, and oil-degraded PET fractions^1,22,23,26,27,31,36,40^.

The scheme in Fig. 13 describes different possible chemical effects of UV and O_2_ oxidation agents on oil/PET chemical transformations and degraded oil/degraded PET surface interactions over time.

In addition, should spin finish oil be unstable to air and light exposure over time, then SFR should best be achieved rapidly post-production of PET fibers and fabrics, or PET storage should be performed more vigilantly in the absence of air or light. Otherwise, finishing oil will chemically transform from desirable hydrophobic protective, lubricating coating states to hydrophilic chemistry via oxidation and/or hydrolysis, and may generate propagating radicals and bonding/solubilization of degraded PET surfaces. Long-term finished PET fiber storage in the presence of degraded oil enhances: (a) generation of fiber surface hydroxyl groups, water absorption and oil/water emulsion formation at fiber surfaces; and (b) hydrolytic PET surface degradation that can then bond or imbibe this oil–water mixture^23,41^.

### Evaluating PET fabrics based on fiber surface nano-mechanical properties

PET surface modifications from aging, storage, SFR or not, grafting or not, and degradation of PET fibers/fabrics were analyzed by AFM (Fig. 9). The surface Young’s modulus distribution reflects relative PET fiber hydrolytic degradation. The highest surface Young’s modulus was found for PET_2018_ after DE-SFR, whereas softer surfaces from PET_2009 and_ PET_1993_ reflected PET-oil interactions and possible water sorption and polymer chain scission prior to grafting^43,44^. More extensive time-dependent degradation/oxidation for SF oil on PET_1993_, with or without polymer grafting, show similar results to deliberate ozonation plus time-dependent aging effects plus polymer grafting shown for PET_2009_. The surface modulus for PET_1993_ does not change further after ozonation or functionalization, consistent with terminal degradation effects on non-grafted PET_1993_ surfaces from degraded oil effects and oxidative PET surface polymer chain scission.

Deleterious effects on PET fibers from finishing oil aging were assumed to derive from:

1. Finishing oil degradation: the spin finish oil used for PET_2009_ and PET_2018_ is derived from modified soybean oil and is FDA-approved for fibers/fabrics dedicated to food packaging and medical devices, but is not inert.
2. Finishing oil degrades, interacts and swells PET_1993_ surfaces in long-term storage (Figs. 1, 4, 5, AFM topography, Fig. 9). SFR oil removal extracts an excessive amount of mass from these aged fiber samples (Figs. 1, 2, 3, 4, 13), indicating that the imbibed oxidized finishing oil plus any degraded PET oil-soluble products are extracted from these oil-swollen PET fabrics under SFR (Fig. 3A).

## Conclusions

Evaluation of the influence of spin-finish oil and storage on PET fabric degradation and fiber surface functionalization with grafted anionic polystyrene sulfonate for PET fabrics used in clinical-grade PET ligament prostheses is reported. Analysis of storage times for finishing oils on PET fabrics prior to SF oil removal by SFR extraction, and the chemical nature of spin finish oil aging on PET substrate properties was used to elucidate unknown relationships between SFR oil extraction from PET over time, and polymer PNaSS grafting efficiency on PET fibers for medical use. Such studies are not reported, yet are important for identifying challenges to fabric shelf-life, storage and processing conditions, and compromising limitations in these polymer medical implant materials.

Results highlight the importance of the following parameters on PET fiber surface quality in storage: (1) the nature of the spin finish oil; (2) the storage time and conditions for spin finished PET fabrics; (3) the choice of SFR solvent for spin finish removal in order to optimize solvent extraction efficiency and further functionalization of PET fibers and fabric surfaces. Cumulative and collective effects of these processes on PET device bulk properties and medical implant performance will be critical to further ascertain.

Our data suggest likely SF oil and PET fiber degradation mechanisms involving finishing oil chemistry and aging (i.e., oxidation in situ) that modify PET-oil interactions over and promote PET surface degradation. Storage conditions facilitate chemical transformations and resulting interactions for degrading spin finish oil with PET, producing modified PET/oil surfaces exhibiting: (1) resistance to SF oil removal by solvent extraction, (2) reduced surface activation and functionalization using PNaSS polymer grafting-from processes; (3) likely chemical and physical surface compromises to PET fibers and fabrics from prolonged interactions with degraded SF oil, and (4) changes in PET fiber surface chemistry and surface mechanical properties. Significantly, the longer the fiber storage time, the more evident the SF oil changes, the more evident the PET fiber degradation, the lower the PET surface PNaSS GR, and the lower the PET surface mechanical integrity.

Different PET surface changes based on spin finish oil, storage time, and grafting-from processing were investigated through AFM analyses. Surface roughness and Young’s moduli for the different PET fiber surfaces were distinct for PET fibers both before and after surface functionalization and correlated with time-dependent production of long-term storage degradation products from both oil and PET surfaces. Spin finish oil extracted from stored commercial fabrics provides clear evidence for formation of hydroxylated fatty acids from SF oil oxidation long-term. Results highlight the importance of the following parameters.

## Supplementary Information

Supplementary Information 1.

## Abbreviations

AFM: Atomic force microscopy
DE: Diethyl ether
DSC: Differential scanning calorimetry
FTIR: Fourier transform infrared spectroscopy
PET: Polyethylene terephthalate
NaSS/pNaSS: Sodium 4-styrene sulfonate/poly(sodium 4-styrene sulfonate)
SFR: Spin finish removal
SEM: Scanning electron microscopy
THF: Tetrahydrofuran
TB: Toluidine blue
UV: Ultraviolet
PET_1993_: Polyethylene terephthalate ligament fibers produced from fiber knitting in 1993
PET_2009_: Polyethylene terephthalate ligament fibers produced from fiber knitting in 2009
PET_2018_: Polyethylene terephthalate ligament fibers produced from fiber knitting in 2018
