# Supplementary material for: Influence of spin finish on degradation, functionalization and long-term storage of polyethylene terephthalate fabrics dedicated to ligament prostheses
Source: Sci Rep. 2021 Feb 19;11:4258. doi: 10.1038/s41598-021-83572-8 (PMC7895958; doi:10.1038/s41598-021-83572-8)
Supplement: Supplementary file 1 — Supplementary Information 1. [file 41598_2021_83572_MOESM1_ESM.pdf]

Supplementary information

Influence of spin finish on degradation, functionalization and long-term storage of polyethylene terephthalate fabrics dedicated to ligament prostheses

Tuan Ngoc Nguyen[1], Andre Rangel[1], David W. Grainger[2], Véronique Migonney[1]

[1]*Chemistry, Structures and Properties of Biomaterials and Therapeutic Agents Laboratory, CSPBAT- UMR CNRS 7244, Université Sorbonne Paris Nord, France*

[2]*Department of Biomedical Engineering, and Department of Pharmaceutics and Pharmaceutical Chemistry, University of Utah, Salt Lake City, UT, USA*

Supporting Information

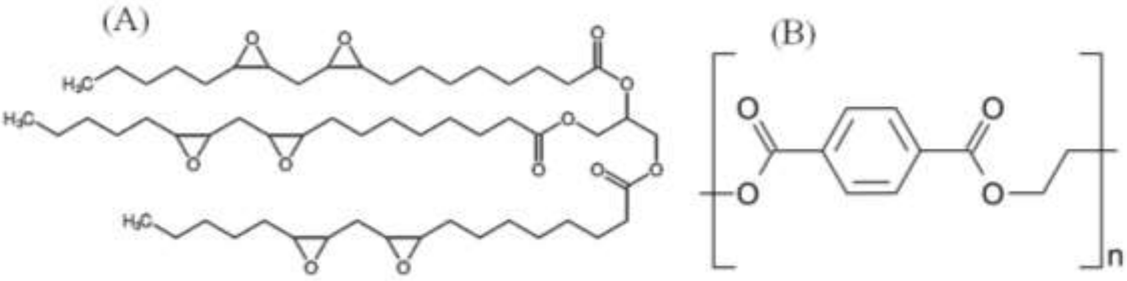

Figure A.1. (A) Structure of epoxidized linolein, a major component of spin-finish epoxidized soybean oil - yellowish viscous liquid [29,34]; **(B)** the structure of polyethylene terephthalate (PET) [39]

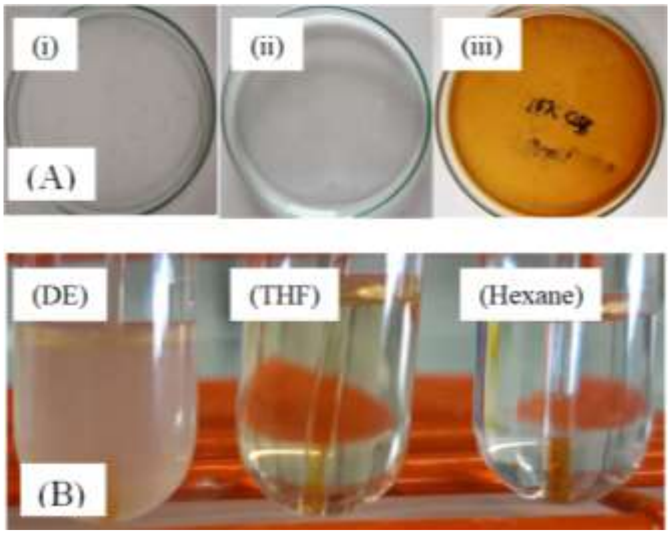

Figure A.2. Spin-finish SF-oil treatment: **(A)** Degradation of SF-oils by UV treatment and thermal treatment: **(i)** virgin SF oil, **(ii)** UV treated SF-oil, **(iii)** thermally treated SF-oil; and **(B)** Solubility of treated oil in diethyl ether (DE), tetrahydrofuran (THF), and n-hexane.

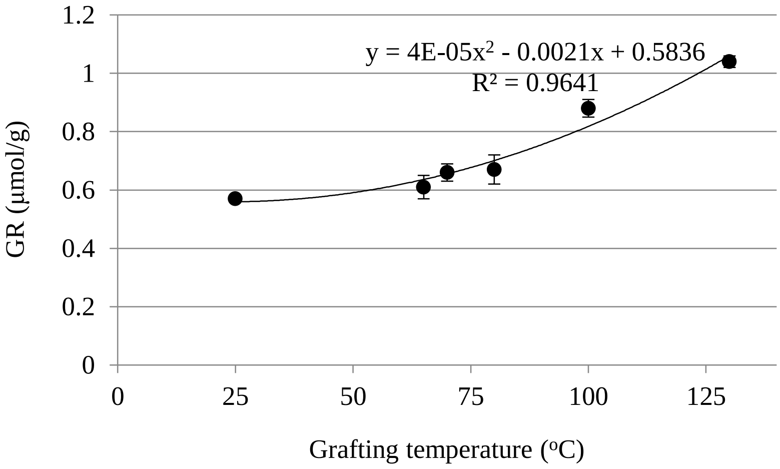

Figure A.3. PNaSS GR of PET_{2009} fabrics at various grafting temperatures: ozonation 10 min at 30°C, grafting for 1h at 25, 65, 70, 80, 100, 130°C (n=3).

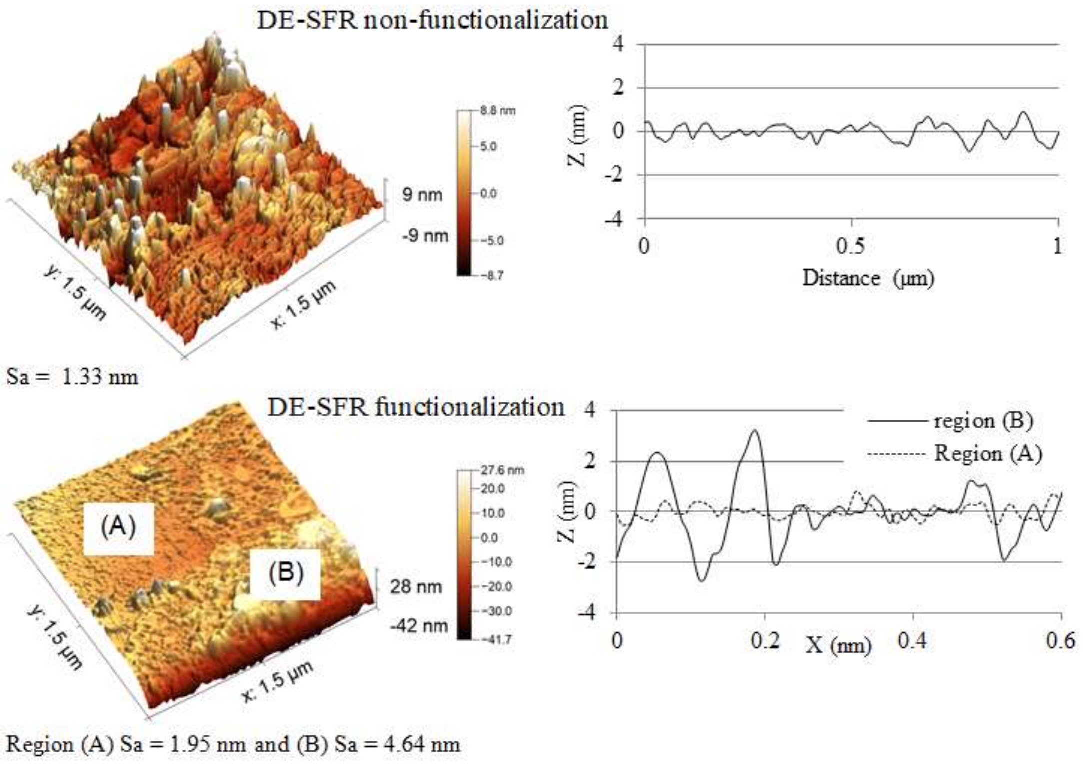

Figure A.4. Topography and roughness of PET_{1993} fibers after (top) DE SFR and (bottom) after DE-SFR + PNaSS grafting.

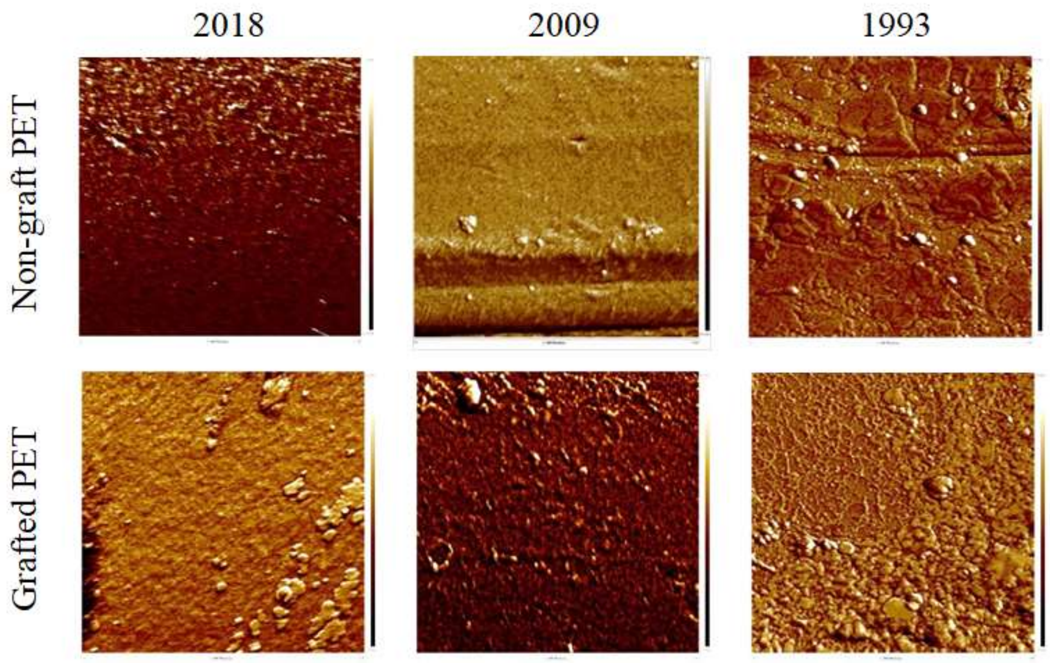

Figure A.5. Surface Young's modulus imaging by Peakforce QNM mapping for spin finish-removed non-grafted and PNaSS-grafted PET fabrics.

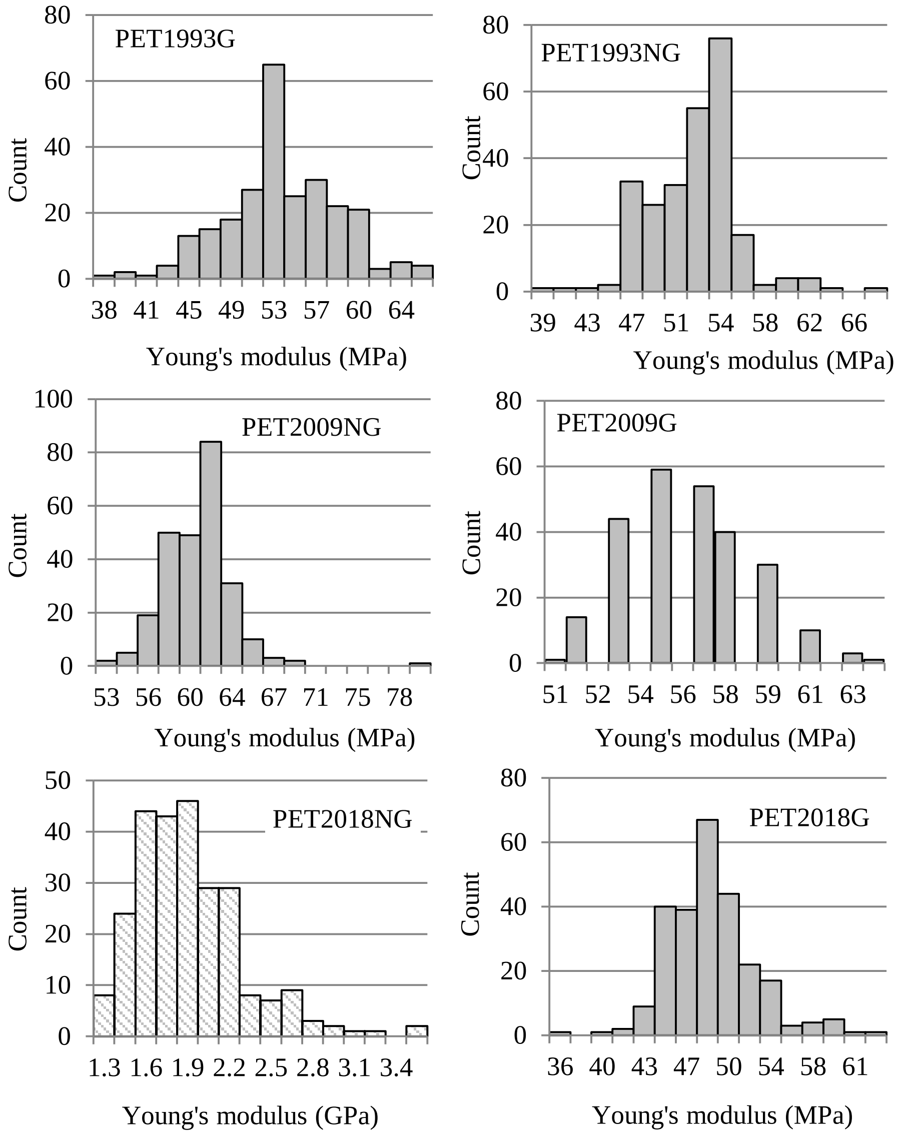

Figure A.6 Young's modulus histogram of PET fibers/fabrics $_{1993,\ 2009,\ 2018}$ non-grafted (**NG**) and PNaSS grafted (**G**). Force Volume program was used with scanning area 500 nm × 500 nm (scan rate = 0.5 Hz).
